# Comprehensive Cohort Analysis of Mutational Spectrum in Early Onset Breast Cancer Patients

**DOI:** 10.3390/cancers12082089

**Published:** 2020-07-28

**Authors:** Mohit K. Midha, Yu-Feng Huang, Hsiao-Hsiang Yang, Tan-Chi Fan, Nai-Chuan Chang, Tzu-Han Chen, Yu-Tai Wang, Wen-Hung Kuo, King-Jen Chang, Chen-Yang Shen, Alice L. Yu, Kuo-Ping Chiu, Chien-Jen Chen

**Affiliations:** 1 Genomics Research Center, Academia Sinica, Taipei 11529, Taiwan; mohitkmidha@sinica.edu.tw (M.K.M.); yfh1202@sinica.edu.tw (Y.-F.H.); chentzuhan@gmail.com (T.-H.C.); chencj@gate.sinica.edu.tw (C.-J.C.); 2 Institute of Biochemistry and Molecular Biology, National Yang-Ming University, Taipei 112, Taiwan; 3 Department of Medical Research, Hsinchu Mackay Memorial Hospital, Hsinchu 300, Taiwan; vul4790127@gmail.com; 4 Institute of Stem Cell and Translational Cancer Research, Chang Gung Memorial Hospital at Linkou and Chang Gung University, No. 5, Fu-Shin St., Kuei Shang, Taoyuan 333, Taiwan; tcfan@cgmh.org.tw (T.-C.F.); logome@gmail.com (N.-C.C.); a1yu@ucsd.edu (A.L.Y.); 5 National Center for High-Performance Computing, Hsinchu Science Park, Hsinchu 300, Taiwan; yutaiwang@narlabs.org.tw; 6 Department of Surgery, National Taiwan University Hospital, Taipei 100, Taiwan; brcancer@gmail.com (W.-H.K.); kingjen@ntu.edu.tw (K.-J.C.); 7 Institute of Biomedical Sciences, Academia Sinica, Taipei 11529, Taiwan; bmcys@ibms.sinica.edu.tw; 8 Department of Pediatrics, University of California in San Diego, San Diego, CA 92161, USA; 9 Department of Life Sciences, College of Life Sciences, National Taiwan University, Taipei 10617, Taiwan

**Keywords:** early onset breast cancer (EOBC), missense mutations, nonsynonymous mutations, somatic mutations, germline mutations

## Abstract

Early onset breast cancer (EOBC), diagnosed at age ~40 or younger, is associated with a poorer prognosis and higher mortality rate compared to breast cancer diagnosed at age 50 or older. EOBC poses a serious threat to public health and requires in-depth investigation. We studied a cohort comprising 90 Taiwanese female patients, aiming to unravel the underlying mechanisms of EOBC etiopathogenesis. Sequence data generated by whole-exome sequencing (WES) and whole-genome sequencing (WGS) from white blood cell (WBC)–tumor pairs were analyzed to identify somatic missense mutations, copy number variations (CNVs) and germline missense mutations. Similar to regular breast cancer, the key somatic mutation-susceptibility genes of EOBC include *TP53* (40% prevalence), *PIK3CA* (37%), *GATA3* (17%) and *KMT2C* (17%), which are frequently reported in breast cancer; however, the structural protein-coding genes *MUC17* (19%), *FLG* (16%) and *NEBL* (11%) show a significantly higher prevalence in EOBC. Furthermore, the top 2 genes harboring EOBC germline mutations, *MUC16* (19%) and *KRT18* (19%), encode structural proteins. Compared to conventional breast cancer, an unexpectedly higher number of EOBC susceptibility genes encode structural proteins. We suspect that mutations in structural proteins may increase physical permeability to environmental hormones and carcinogens and cause breast cancer to occur at a young age.

## 1. Introduction

Early onset breast cancer (EOBC) diagnosed at age ~40 or younger is associated with a poorer prognosis, higher recurrence rate and higher mortality rate than breast cancer (BC) diagnosed at later ages [1,2,3]. Overall, there are over one million (e.g., 1.38 million in 2008 alone) new breast cancer cases each year worldwide [4,5]. Among these cases, approximately 4%–14% are EOBC: ~6.6% in American women [6], ~12% in Taiwanese women [7] and over 13% in Singaporean women [8]. While late-onset breast cancer (LOBC) is becoming more manageable, EOBC has continued to increase steadily in many countries over the past few decades and demands intensive investigation [3,6,9,10,11]. Breast cancer encompasses a broad spectrum of heterogeneous malignancies resulting from polygenic susceptibility. Based on the pattern of gene expression and histopathologic staining [12,13,14], previous studies have classified breast cancer into several clinically relevant subtypes, each of which is more or less associated with epidemiological risk factors and clinical responses [11,15,16].

A broad spectrum of risk factors has been associated with breast cancer. These risk factors include genetic predisposition (e.g., familial history and physiological history), lifestyle (e.g., dietary, living and physical activity habits) and environmental factors (e.g., environmental carcinogens and hormones). These risk factors may vary significantly between ethnic groups and racial origins [4,11,17], making the attempt to elucidate the underlying etiopathologic causes a great challenge; thus, a broad range of analyses targeting as many potential risk factors as possible are strongly desired.

This study aims to obtain comprehensive insight into EOBC. We surveyed somatic mutations, copy number variations (CNVs) and germline mutations in 90 patients to reveal the etiopathologic factors involved in the tumorigenesis of EOBC. Through the comparison of missense mutations between white blood cell (WBC)–tumor pairs, we examined the association of these molecular alterations with biologic pathways and clinical subtypes. The results indicate that a significantly higher fraction of EOBC susceptibility genes encode extracellular structural proteins compared to breast cancer susceptibility genes as a whole.

## 2. Results

### 2.1. Structure of the EOBC Cohort

The EOBC cohort included all five subtypes (Table 1). The median age of the patients was 37, and the tumor stages ranged from Ia—IVb, with the largest number of patients at stages IIa and IIb. Eleven patients had a family history of breast cancer, including 2 sisters (IDs 7768 and 7942), who will be further discussed below.

The base coverage of the WGS libraries ranged between 30.6–44.2-fold, while that of the WES libraries ranged between 40–167-fold (Supplementary Table S1).

### 2.2. Somatic Mutation Analysis

A total of 17,175 somatic mutations distributed in 7475 protein-coding genes were identified. These mutations belonged to many categories, among which missense mutations constituted the majority (91.7%) (Supplementary Table S2).

To facilitate further discussion, a table in which patient clinical data were integrated with somatic mutation-associated information, especially for *TP53*, *BRCA1* and *BRCA2*, was generated (Supplementary Table S3) and the issues discussed in the remainder of this section are deduced from the information provided in table.

The *TP53* and *PIK3CA* together with three other randomly selected genes were validated by Sanger sequencing using patient BC0145 as an example (Supplementary Figure S1). The mutations in genomic locations found in tumor tissues were confirmed for all genes tested.

As frequently observed in breast cancer, a small portion of patients may exhibit distinctly high number of genes with somatic mutations. Here, among the 90 patients, 6 patients were found to have somatic mutations in more than one thousand genes (ranging from 1237–1926 genes, avg. 1520 per patient); these were categorized as high-mutation (HM) patients (Supplementary Figure S2). The remaining patients exhibited somatic mutations in 7–402 (avg. 63) genes and were categorized as low-mutation (LM) patients.

A striking characteristic of the HM patients was that all of them had somatic mutations in the *BRCA1* gene, and half of them also had somatic mutation(s) in the *TP53* and/or *BRCA2* gene (Supplementary Table S3). All three genes encode tumor suppressors involved in the DNA repair checkpoint and are among the most common susceptibility genes in breast cancer. The influence of these genes in determining the HM phenotype was further suggested by sisters 7768 and 7924 (both luminal B/Her2+). The elder sister, 7768, did not exhibit any mutation in any of these genes and belonged to the LM group, while the younger sister, 7924, had mutations in all three genes and belonged to the HM group. (More information regarding these sisters is shown in later section entitled “Case study of sisters in the cohort”.) Notably, the HM phenotype did not seem to correlate with clinical stage or with any particular subtype.

**Figure 1:**
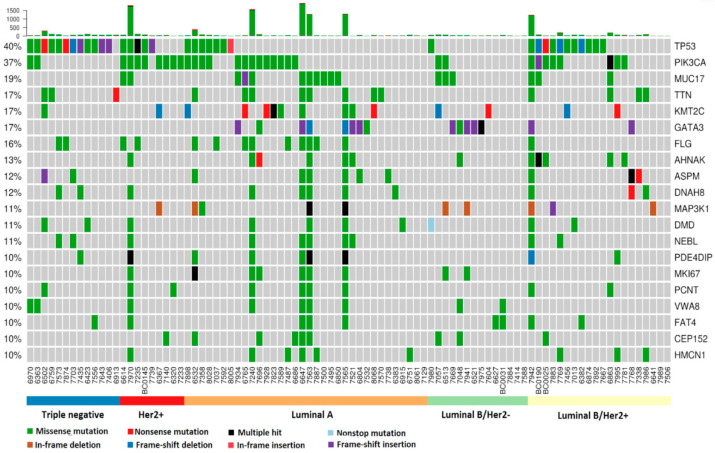
Top genes with somatic mutations with at least 10% prevalence among EOBC patients. The number on the top of each bar represents the number of mutations in a patient. High-mutation (HM) patients (6 total) can be distinctly identified. In the central panel, patients’ mutated genes are labeled with various colors, each representing a particular type of somatic mutation. The names of the mutated genes are listed on the right side of the panel, while the corresponding prevalence is shown on the left side of the panel.

The genes that were most commonly affected by somatic mutations were similar to those previously reported in breast cancer (Figure 1). *TP53* and *PIK3CA* were distinctly ranked as first and second, with prevalences of 40% and 37%, respectively, followed by extracellular structural protein-coding genes *MUC17* (19%), *TTN* (17%) and *FLG* (16%), together with the transcription factors *KMT2C*/*MLL3* (17%) and *GATA3* (17%).

Notably, *TP53* somatic mutations showed a 92% prevalence among triple-negative patients (followed by a 55% prevalence in luminal B/Her2+ and 56% in Her2+), while *PIK3CA* somatic mutations showed an 89% prevalence among Her2+ patients (Supplementary Table S4). Interestingly, *GATA3* mutations were mainly associated with the luminal B/Her2- (36%) and luminal A (24%) subtypes.

*MUC17* encodes mucin 17, which is a highly expressed membrane-bound glycoprotein in intestinal epithelial cells that provides protection for the cells where it is expressed. The *TTN* gene encodes titin or connectin, which functions as a molecular spring by providing elasticity to muscle. Mutations in titin proteins result in the weakening of muscle and are responsible for heart disease and muscular dystrophy. The *FLG* gene encodes the filaggrin protein, which undergoes proteolysis to generate filaggrin monomers. By integrating into the cellular membrane, filaggrin monomers strengthen the skin barrier.

### 2.3. Comparison of Top Genes with Somatic Mutations in Taiwanese EOBC Cohort to Top Genes in EOBC and Non-EOBC Groups from Other Breast Cancer Cohorts

To gain more insight into the common and unique genic features of Taiwanese EOBC patients, we compared most prevalent genes in our EOBC cohort to their counterparts in EOBC and non-EOBC groups generated by combining 6 non-Taiwanese (external) cohorts.

Among the six non-Taiwanese cohorts, most patients were non-EOBC cases (50.93%–92.78%), while EOBC cases ranged only between 7.22%–49.07% (Supplementary Table S5). Overall, 690 (13.97%) and 4248 (86.03%) patient’s mutation and clinical data (including Taiwanese EOBC cohort) were analyzed for characterization of EOBC and non-EOBC, respectively.

For the publicly available breast cancer datasets, most of the frequently mutated BC susceptible genes were observed in both EOBC and non-EOBC groups, although the mutation frequencies were significantly different for certain genes (Supplementary Table S6). Mutations in *TP53* and *PIK3CA* genes were predominant among both EOBC and non-EOBC patients in the public datasets. Mutation frequency of *TP53*, *GATA3* and *ESR1* were higher in EOBC cases than non-EOBC cases. Mutations in *PIK3CA*, *CDH1*, *KMT2C* and *MAP3K1* were more frequently observed in non-EOBC cases.

**Figure 2:**
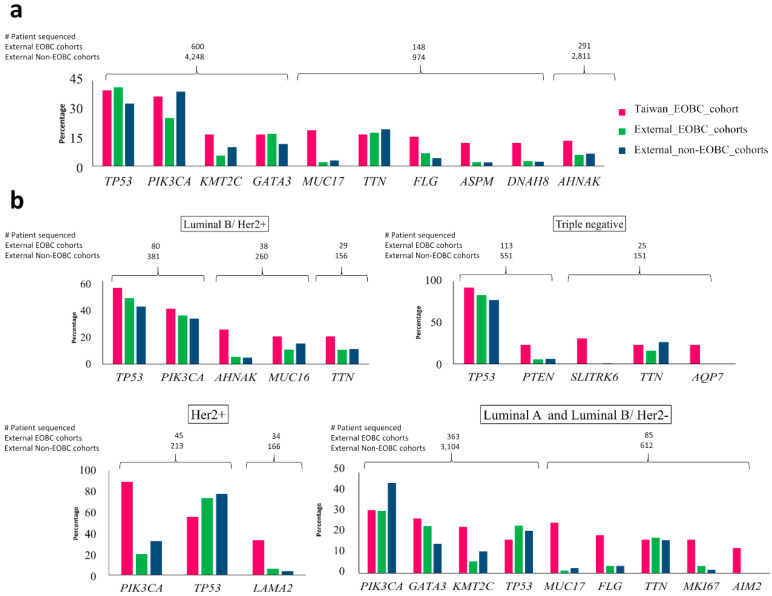
Comparison of top genes with somatic mutations identified in Taiwanese EOBC cohort to the pooled EOBC and pooled non-EOBC groups in other cohorts. (**a**) Patients from external cohorts were divided into EOBC and non-EOBC patients and then pooled together to form EOBC and non-EOBC groups and compared with Taiwanese EOBC counterparts; (**b**) subtype-based comparison of top genes with somatic mutations in Taiwanese EOBC-to-EOBC and non-EOBC counterparts in external cohorts. For the comparison, EOBC and non-EOBC groups were further divided into subtypes. The most prevalent genes in each subtype of Taiwanese EOBC were compared to their counterpart genes in each subtype of EOBC and non-EOBC groups from external cohorts. For external cohorts luminal A and luminal B/Her2- subtypes were indistinguishable due to lack of information on ki67. These two subtypes from the Taiwanese EOBC cases were combined and compared with external cohorts. Percentage of patients with mutation in each gene is shown by vertical bars and the number of patients sequenced for each gene in external EOBC and non-EOBC cohorts is shown at the top of that gene.

We compared mutation frequencies of the top 10 genes with somatic mutations in the Taiwanese EOBC cohort to mutation frequencies of these genes in the pooled EOBC and non-EOBC patients (Figure 2, panel a).

The mutation frequency of *TP53* was similar in Taiwanese EOBC and pooled EOBC (40% and 41.5%, respectively), but higher than that of pooled non-EOBC (32.9%). A similar pattern was observed for *GATA3* (Taiwan EOBC, 16.7%; pooled EOBC, 17%; pooled non-EOBC, 11.6%). *PIK3CA* was mutated in 25.3% of pooled EOBC patients, 36.7% of Taiwanese EOBC patients and 39.3% in pooled non-EOBC patients. The mutation frequency of *KMT2C* was found to be highest in Taiwanese EOBC patients (16.7%), followed by pooled non-EOBC (10%) and pooled EOBC patients (5.5%). Mutation frequencies of *MUC17* (19%), *FLG* (15.6%), *AHNAK* (13.3%), *ASPM* (12.2%) and *DNAH8* (12.2%) were distinctly higher in Taiwanese EOBC patients than that of pooled EOBC and non-EOBC groups from other cohorts.

We then divided all pooled EOBC group and non-EOBC group into subtypes and compared the prevalences of the top genes in each subtype (Figure 2, panel b). For the Taiwanese EOBC cohort, luminal B/Her2+ subtype had *TP53* (55%)*, PIK3CA* (40%)*, AHNAK* (25%)*, MUC16* (20%) and *TTN* (20%) as the most prevalently mutated genes; the triple-negative subtype had *TP53* (92%)*, SLITRK6* (31%)*, PTEN* (23%)*, TTN* (23%) and *AQP7* (23%) as the most prevalently mutated genes; and the Her2+ subtype had *PIK3CA* (88.9%)*, TP53* (55.6%) and *LAMA2* (33.3%) as the most prevalently mutated genes.

Through the comparison, we observed that in the luminal B/Her2+ subtype, *TP53*, *PIK3CA*, *AHNAK*, *MUC16* and *TTN* have similar mutation frequencies among pooled EOBC and pooled non-EOBC patients from external cohorts and that their mutation frequencies in the Taiwanese EOBC cohort are slightly higher. We observed similar mutation frequencies for *TP53*, *PTEN* and *TTN* in the triple-negative subtype of pooled EOBC and pooled non-EOBC groups from external cohorts, but their mutation frequencies were higher in the Taiwanese EOBC cohort. In the Her2+ subtype of Taiwanese EOBC patients, mutation frequencies of *PIK3CA* and *LAMA2* (88.9% and 33.3%, respectively) were higher than that of pooled EOBC (20% and 5.9%, respectively) and pooled non-EOBC groups (32.4% and 3.6%, respectively) of other cohorts, whereas the *TP53* mutation frequency (55.6%) was lower than that of pooled EOBC (73.3%) and pooled non-EOBC (77.5%) groups. In the Her2+ subtype of external cohorts, *TP53* was the most prevalently mutated in both pooled EOBC and pooled non-EOBC patients, whereas in the Her2+ subtype of the Taiwanese cohort, *PIK3CA* was most prevalently mutated. In “luminal A and luminal B/Her2-” subtypes with ER+/PR+/Her2- status, mutation frequency of *PIK3CA* was lower in all EOBC patients than that of non-EOBC patients, while *GATA3* was higher.

Mutation frequencies of the top 10 genes in the Taiwanese EOBC cohort were compared with EOBC and non-EOBC groups of each external cohort individually. For *TP53*, variations in mutation frequencies among individual EOBC cohorts were higher than non-EOBC cohorts (Supplementary Figure S3). Variations in mutation frequencies of *PIK3CA* and *TTN* were moderately high in EOBC compared to non-EOBC patients across all cohorts, whereas variations in mutation frequencies of the other seven genes among EOBC and non-EOBC groups were similar across all cohorts.

We also compared the survival and disease/progression free survival along with the molecular characteristics of pooled EOBC and pooled non-EOBC groups. The survival of EOBC and non-EOBC was analyzed over a 420-month period. Patients from the non-EOBC group exhibited poor survival probabilities. Age, being an important risk factor for non-EOBC patients, corresponds to poor survival. Disease/progression-free survival time of patients from the pooled non-EOBC group was significantly higher than that of the pooled EOBC group over a 220-month period (Supplementary Figure S4). The hazard ratio of EOBC was 1.96 (confidence interval 1.05–3.51, *p*-value 0.0321) indicating an almost two times higher risk of cancer progression among the EOBC patients than among the non-EOBC patients.

### 2.4. Association of Somatic Mutations with Family History of Breast Cancer

There were 11 patients in the cohort with a family history of breast cancer. Patients with and without a family history of breast cancer seemed to differ in the preferentially acquired somatic mutations. Patients with a family history were more likely to have somatic mutations in *TP53* (56%), *TTN* (36%) and *GATA3* (27%), while patients without a familiar history were likely to have somatic mutations in *PIK3CA* (39%), *TP53* (38%) and *MUC17* (20%). However, the data may not be representative because of the small population size for patients with a family history.

### 2.5. Copy Number Variation Is Associated with Subtypes

CNV analysis revealed an association of CNVs with subtypes, each of which was in turn associated with CNVs of particular proto-oncogenes and tumor suppressor genes in the affected regions (Supplementary Table S7). Most evidently, the Her2+ subtype was associated with copy number increases in the proto-oncogenes *MLLT6*, *TBC1D3* and *TAF15,* but not in any tumor suppressor, while the luminal A subtype was associated with copy number increases in the proto-oncogenes *GNAS*, *TRIM27*, *MDM2*, *MCF2 L* and *RARA,* but in no tumor suppressor genes.

### 2.6. Germline Mutations

**Figure 3:**
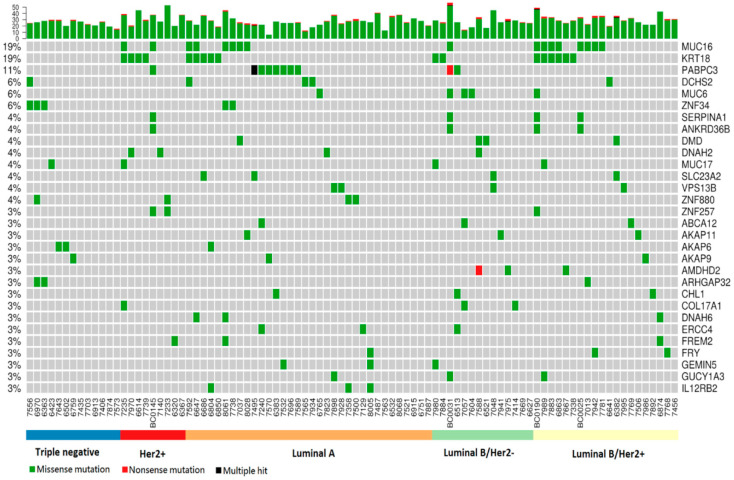
Genes with germline mutations among the 90-patient EOBC cohort. (Top) Each bar represents the number of mutations for a patient; (**Central panel**) Patient mutated genes are labeled with various colors, each representing a particular type of germline mutation. The names of the mutated genes are listed on the right, with the corresponding prevalence shown on the left side.

A total of 2690 germline mutations, affecting 2170 genes, were identified. Among these mutations, 2595 were missense mutations, while 95 were nonsense mutations. The genes with the most germline mutations included *MUC16* (19% prevalence), *KRT18* (19%), *PABPC3* (11%), *DCHS2* (6%), *MUC6* (6%) and *ZNF34* (6%) (Figure 3). Many of these genes encode extracellular and/or structural proteins.

*MUC16* encodes mucin 16, an o-glycosylated protein that forms a protective mucous layer on the surface of epithelia. *KRT18* (keratin 18) encodes keratin 18, a type I intermediate cytoskeleton filament. *PABPC3* encodes poly(A)-binding protein, cytoplasmic 3, involved in mRNA metabolism and translational initiation. *DCHS2* encodes dachsous cadherin-related-2, a large protein containing a large number of cadherin domains.

### 2.7. Pathway Analysis

Combinatorial pathway analyses of subtypes by integrating somatic mutations and germline mutations revealed a number of interesting pathways (Table 2). Genes in small cell lung cancer and focal adhesion pathways were commonly altered across all five subtypes. Mutations identified in Her2+ patients were enriched in osteoclast differentiation, sphingolipid-signaling and thyroid hormone-signaling pathways. ECM receptor interactions and PIK3-Akt-signaling pathways were altered in luminal A and both of the luminal B subtypes. Interestingly, in both luminal B/Her+ and luminal B/Her2- subtypes, ABC (ATP-binding cassette) transporter pathway was altered exclusively. Three pathways (namely small cell lung cancer, focal adhesion and endometrial cancer pathways) were altered in triple-negative subtype patients, which were common with other subtypes. The luminal B/Her2- subtype exhibited the most diverse affected pathways. Overall, the ABC transporter pathway, followed by ECM–receptor interaction and the focal adhesion and PI3K–Akt pathways, were the most significantly affected pathways among the patients in the Taiwanese EOBC cohort as a whole.

### 2.8. Case Study of Sisters in the Cohort

To better understand whether family history plays a pathologic role that is predictive of EOBC tumorigenesis, we paid special attention to the sister pair with a focus on the common and different features between their germline backgrounds. These sisters (IDs 7768 and 7942) were diagnosed with breast cancer at ages 29 and 28, respectively and both belonged to the luminal B/Her2+ subtype and presented very similar CNV patterns (Supplementary Table S8).

In terms of germline mutations, patient 7768, belonging to the LM group, exhibited 31 mutated sites distributed in 31 genes, while patient 7942, belonging to the HM group, exhibited 37 mutated sites distributed in 36 genes. Nineteen sites distributed in 18 genes were shared by these two women, including a site in the FRY gene that showed a 3% prevalence among all EOBC patients (Supplementary Figure S5). However, they exhibited very different somatic mutations and mutations in *TP53*, *BRCA1* and *BRCA2* found in patient 7942 seemed to have been acquired after birth.

## 3. Discussion

We surveyed germline and somatic missense mutations as well as copy number variations in the EOBC cohort of Taiwanese women. Comparison of our EOBC with external EOBC and non-EOBC cohorts showed that *TP53* had the highest mutation frequency in EOBC across all cohorts, indicating that the *TP53*-mutation rate was significantly higher in EOBC patients. On the other hand, *PIK3CA* had the highest rate of mutation in non-EOBC patients. This phenomenon was consistent across all cohorts analyzed [18]. In addition, the Taiwanese population has the highest prevalence of *TP53* among the Asian populations that have been investigated. In a South Korean cohort of 229 patients with ER+ and Her2- breast cancer, *TP53* was found mutated in 10% of the patients [19], compared to 16.7% in ‘luminal A and luminal B/Her2- combined’ in our cohort. In a study of 116 triple-negative patients from Thailand [20], 76% were found to carry *TP53* mutations, comparing to 92% in the same subtype in our cohort. Recapitulating, the high prevalence of mutations in *TP53* implies a distinct molecular characteristic of EOBC and associated subtypes.

Family history was known to be associated with higher risk of EOBC than LOBC [21]. In American women, patients with family history of breast or ovarian cancer in first degree relatives had higher risk for EOBC (4.9% to 10.0%) and lower risk for LOBC (2.0% to 2.1%) [21]. Inherited genetic risk factors such as *BRCA1* and *BRCA2* mutations are observed more often among breast cancer patients, but are not the sole causal factors [22,23,24]. In a study of 1987 Dutch women with or without breast cancer, family history of breast cancer in first degree relatives was found to be one of the prominent risk factors for EOBC [25]. In our study, 11 EOBC patients had family history of breast cancer up to second degree of relatives. Although *BRCA1*/2 or *TP53* mutations among the family members have been reported as a risk factors of EOBC, our study was limited due to the lack of background genetic information of family members for comparison. Several other studies related to breast cancers did not solely focus on EOBC in particular [18,26,27,28,29], while studies with a focus on EOBC either analyzed a particular subtype or preselected genes, and the cohort may be too small and thus not representative [19,20,23,30,31,32,33,34]. Our study included 5 major subtypes of breast cancer in young women (less than 40) and to the best of our knowledge, this is so far the only comprehensive study of EOBC in Taiwanese women. Similar to the previous reports by other groups, somatic mutations in genes such as *TP53*, *PIK3CA* and *GATA3* are associated with EOBC [19,20,30,33], while high prevalences in somatic mutations were observed in certain genes such as *KMT2C*, *FLG*, *MUC17* and *ASPM* in our cohort.

It was astonishing to observe an extremely large number of mutations in structural protein-coding genes in the studied group. This phenomenon was shown by multiple lines of evidence. First, the top 2 genes with germline mutations were *MUC16* and *KRT18*, both of which exhibited a 19% prevalence and encoded structural proteins. In addition, more mucin-coding genes, including *MUC6* and *MUC17*, were highly mutated. Second, compared to conventional breast cancer, somatic mutations in *MUC17*, *FLG* and *NEBL* were of much higher prevalence among EOBC patients. Third, these observations were further strengthened by the pathway analysis, which showed that the focal adhesion and ECM–receptor interaction pathways were the most prevalent affected pathways.

The *MUC16* gene contains multiple occurrences of the SEA domain (Sea urchin sperm protein, enterokinase, agrin) and its protein is thought to play a role in forming a protective layer for epithelial cells from pathogens and foreign particles/ligands [35]. In our analysis, we reported germline variations in this gene at regions where copies of the SEA domain are located. These variations are nonsynonymous in nature and may change the structural integrity of the barrier. Several studies have reported on the role of *MUC16* in different types of cancers including breast cancer [36,37,38,39,40]. As these alterations are germline variations, we hypothesize that, in breast cells they may have allowed the uptake/release of cell-signaling molecules leading to initiation/blockage of signaling pathways, subsequently triggering tumor development and progression at an early age in these women. Apart from *MUC16* which has germline variation present in 19% of the EOBC patients, *KRT18* and *PAPBC3* genes were having germline variations in 19% and 11% of patients, respectively. Interestingly, the latter two variations were mutually exclusive. *KRT18* is a known triple negative breast cancer marker and has also been reported to play a role as oncogene in colorectal cancer [41,42]. *PABPC3* was found to have germline mutation in a breast cancer cohort of Tunisian females [43].

Extracellular and membrane-bound structural proteins form an occlusive barrier to protect an organism from various physical and chemical impacts from the environment. Based on these observations, we hypothesize that mutations in these structural proteins may result in a leaky epidermis and, thus, weaken the physical barrier, which would become more permeable to environmental toxins such as environmental carcinogens or hormones. The enhanced accumulation of toxins within breast cells will increase somatic mutations and initiate tumorigenesis at an early age. In fact, this possibility has been well documented for the filaggrin protein, encoded by the *FLG* gene [44], and functional defects resulting from loss-of-function mutations of *FLG* have been found to be a major risk factor for eczema [45,46]. Thus, besides driver mutations and passenger mutations acting in the downstream of tumorigenesis, mutations in extracellular protein-coding genes may act in the upstream and failure in gatekeeping is responsible for tumorigenesis developing at a young age. The role of gatekeeper may be even more important for the triple-negative subtype, in which ER, PR, Her2 receptors are absent or expressed at extremely low levels.

It is also noteworthy that some of the highly mutated structural genes, such as *TTN* and *MUC17*, encode very large proteins, and one may suspect that high mutation rates in these genes may simply be due to size effect. In fact, both *TTN* and *MUC17* have been directly or indirectly recognized as tumor-associated genes. For example, although most commonly known as a molecular spring by providing elasticity to muscle, *TTN* was found to associate with chromosomes to provide elasticity [47]. With close association with chromosomes, it is reasonable to speculate that mutations in *TTN* may cause mutations in the genome. Direct evidence was provided by Tan and colleagues. In their comprehensive survey of 23 cancers in COSMIC data, *TTN* was ranked second highest in terms of numbers of somatic mutations [48]. Furthermore, the large gene size of *TTN* was noticed in a report by Greenman and colleagues. In their study, *TTN* was ranked as the single most prevalent gene among 518 protein kinase genes in 210 different human cancers associated with an extremely high density of cancer driver mutations [49]. On the other hand, although mucins are essential components of the mucous layer in extracellular space, mucins were also known to be involved in inflammation and cancer [50], while Mucin 17 was further found to inhibit the progression of human gastric cancer [51].

Since tumor tissue availability is limited and varies with surgical requirements for the patient or size of tumor, it is hard to acquire samples for NGS and validation of mutations. In our analysis, we were able to validate mutations in the tumor of one of the patients. Our analysis, although limited by sample size, still correlates with external cohorts and provides important insights into EOBC in Taiwanese women.

## 4. Materials and Methods

### 4.1. WGS: Tissue Sample Collection, DNA Preparation and Whole-Genome Sequencing

Genomic DNA samples from WBC-tumor pairs from 4 patients (BC0145, BC0190, BC0025 and BC0031) ages of < 40 years were prepared at the Institute of Stem Cell and Translational Cancer Research (ISCTCR) at Chang Gung Memorial Hospital, Taiwan and stored at −80 °C according to routine laboratory practice. All genomic DNA samples were extracted by using the Gentra Puregene Blood kit (Qiagen, cat# 158445) and sequenced using the HiSeq X Ten platform (Macrogen, Inc., South Korea) with 150 × 150 bp paired-end (PE) reads.

### 4.2. WES: Patient Recruitment, DNA Preparation and Whole-Exome Sequencing

A total of 86 female EOBC patients were recruited for the study between 2005 and 2010. All patients were diagnosed at age 41 or younger at National Taiwan University Hospital (NTUH). Eleven of them had a family history of breast cancer (mother or sister). Paired peripheral blood samples (buffy coats) and tumor tissues were collected during surgery, and tumor tissue samples were examined by pathologists at NTUH.

Genomic DNA from the WBC samples was isolated immediately after blood collection and stored at −20 °C, while the tumor tissues were stored at −20 °C until DNA isolation. Genomic DNA from the tumor tissues was extracted using the DNeasy blood and tissue kit (Qiagen) and quantified by using the Qubit dsDNA BR assay kit (Invitrogen). Library preparation and exome enrichment were performed using the SureSelect human all exon system (Agilent Technologies) following the manufacturer’s instructions for Illumina PE sequencing. Briefly, the DNA sample was sheared into 150–200 bp fragments. By using the paired-end sample preparation kit, the 5′-ends of the fragments were blunt ended and ‘A’ bases were added to the 3′ ends. Index-specific PE adaptors were then ligated to both ends of the fragments. After removing the unligated adaptors, the ligated fragments were PCR-amplified using an Illumina InPE1.0 primer and a SureSelect indexing precapture primer. The quality and quantity of the purified samples were assessed by using an Agilent 2100 Bioanalyzer. For hybridization, the samples were processed with the Agilent SureSelect automated library prep and capture system. PE sequencing was conducted by using the Illumina genome analyzer IIX (GAIIX) system (Illumina) at 75 × 75 bp.

### 4.3. Subtype Classification

Patients were classified based on the expression of the ER (estrogen receptor), PR (progesterone receptor), Her2 (human epidermal growth factor receptor 2) and Ki67 proteins [52,53]. Protein expression data were provided by the NTUH pathology lab. Immunohistochemical staining of proteins was performed on formalin-fixed, paraffin-embedded tissue sections and double-read by two pathologists. For the assessment of ER and PR expression, samples with >10% positively stained nuclei were defined as positive. Her2 expression was scored as 0, 1+, 2+ or 3+ based on the percentage and intensity of staining. A score of 0 or 1+ was classified as negative and a score of 3+ was classified as positive. Samples with a score of 2+ were examined by fluorescence in situ hybridization (FISH), and those showing positive staining were classified as Her2-positive (Her2+). The cutoff value for the Ki67 protein was staining of 14% of cells. According to the immunohistochemical profiles, these tumors were classified into five subtypes: (1) luminal A (ER+ and/or PR-positive (PR+), Her2-negative (Her2-), Ki67 < 14%); (2) luminal B/Her2- (ER+ and/or PR+, Her2-, Ki67 ≥ 14%); (3) luminal B/Her2+ (ER+ and/or PR+, Her2+, any Ki67); (4) Her2+ - non-luminal (ER-, PR-negative (PR-) and Her2+); and (5) Triple-negative (ER-, PR- and Her2-).

### 4.4. Sequence Data Analysis Workflow

Raw reads from WGS were filtered to obtain quality reads. The procedure included (1) mapping all reads to the PhiX genome using Bowtie2 and then selecting unmappable reads using seqtk; (2) removing Illumina sequencing adapters using Cutadapt; (3) removing low-quality bases from the 5′- and 3′-ends using PRINseq with QV30 as the cutoff; (4) removing reads with at least two ambiguous bases using PRINseq; (5) selecting reads with ≥ 30 bases using PRINseq; (6) selecting quality reads using the NGS QC toolkit [54], (QV20, 70%); and (7) obtaining paired-end and single-end (SE) reads for further analysis. Quality reads were then mapped against human genome assembly hg19 using the Burrows wheeler aligner (BWA) version 0.7.5a [55]. Duplicates were marked using the MarkDuplicates module of PICARD tools (v1.98). The standard GATK pipeline [56], which includes RealignTargetCreator, IndelRealigner, BaseRecalibrator and PrintReads, was used to process bam files before variant calling.

### 4.5. Somatic Mutation Analysis

Paired WBC and tumor sequence data were compared by using MuTect2 (GATK v4beta) with the default parameters to identify somatic mutations, followed by region- and feature-based annotations to identify somatic variants using ANNOVAR v2016Feb01 [57]. The Maftools program in R was used for further analyses, such as plotting mutation status.

### 4.6. Validation of Mutations

To validate mutations, specific PCR primers flanking the mutation sites of five genes were designed and used to generate PCR fragments containing the mutation sites in patient BC0145. PCR fragments were cloned into the pZBack vector (Tools Biotechnology) and the constructs were used to transform *E. coli* DH5-Alpha competent cells, which were then cultured in selective agar plates. Colonies were picked and cultured in aqueous media. Plasmids were isolated with mini-preps and sequenced with Sanger sequencing.

### 4.7. Cross-Comparison of Top Genes with Somatic Mutations between the EOBC Cohort and Other Breast Cancer Studies

To assess the molecular differences in EOBC and non-EOBC individuals, we collected six publicly available breast cancer datasets, which contain mutation and clinical information, from cBioPortal [58,59] and analyzed these datasets in a systematic manner. These datasets were obtained from 5975 samples including (1) 1918 samples from the breast cancer database [26]; (2) 2509 samples from METABRIC [27,28]; (3) 103 samples from the breast invasive carcinoma database [5]; (4) 100 samples from the Sanger Institute [29]; (5) 1108 samples from the TCGA provisional database; and (6) 237 samples from the metastatic breast cancer project. Patients without gender and/or age information, together with male patients, were excluded. Female patients were dichotomized into two groups on the basis of age at diagnosis, and patients ≤41 years were categorized as EOBC and patients above 41 years as non-EOBC.

For the comparison of somatic mutation patterns between EOBC and non-EOBC patients, we first analyzed somatic mutations for all these patients to identify EOBC- or non-EOBC-specific genes and then compared the prevalences of the 10 genes most susceptible to somatic mutations in the Taiwanese EOBC cohort to that in all other cohorts, either on individual or pooled basis for EOBC and non-EOBC separately. Disease/progression free survival time was defined as the time from diagnosis until first occurrence of relapse, progressive disease, secondary cancer or death or, if none occurred, until last contact. Patients from external cohorts were dichotomized into EOBC and non-EOBC groups based on age (<41 years) and survival probability was estimated by Kaplan–Meier survival curves. The relative risk of disease/progression free survival of EOBC was estimated by univariate Cox-proportional hazard regression analysis.

We further divided patients from all datasets into subtypes, based on the criteria of the St. Gallen subtypes classification mentioned previously in the “subtype classification” section. Since most cohorts lacked Ki67 information, classification of luminal A and luminal B/Her2- was not feasible, so we combined these two subtypes into “luminal A and luminal B/Her2-, with receptor status ER+/PR+/Her2-” and analyzed them together with luminal B/Her2+, Her2+ and triple-negative subtypes based on ER, PR and Her2 status. These subtypes were further compared for EOBC and non-EOBC patients combined.

### 4.8. Germline Mutation Analysis

GATK HaplotypeCaller was used to identify germline mutations in WBC samples and annotations were added using ANNOVAR. To select novel germline mutations that may potentially be associated with EOBC, we compared our data with public databases. First, we checked for these mutations in the Taiwan Biobank (database for SNP information from 1000 normal Taiwanese people, https://taiwanview.twbiobank.org.tw/index). Allele frequencies (AFs) were calculated for each germline missense mutation in 90 patients and compared with the AF at the same location in the Taiwan Biobank using the chi-squared test. Genomic locations with significant differences (*p*-value ≤ 0.05) between the sample and Biobank populations are reported in the results. Germline mutations were also checked with the dbSNP and COSMIC databases. Those mutations not present in dbSNP were retained for further analysis.

### 4.9. Copy Number Variation Analysis

Bam files for WBCs and tumors were further processed with GATK modules (CalculateTargetCoverage, CombineReadCounts, CreatePanelOfNormals, NormalizeSomaticReadCounts, PerformSegmentation and CallSegments) to obtain coverage information. We analyzed the CNV profiles of 90 EOBC patients by grouping them according to molecular subtypes to obtain subtype-specific alterations. GISTIC2.0 was used to call targets with somatic copy number alterations in each subtype [60].

### 4.10. Pathway Analysis for Germline and Somatic Mutations

Six lists of genes were prepared for pathway analysis, one for each of the five subtypes and one for the whole cohort. Each list comprised genes with germline mutations and genes with somatic mutations. The following criteria were followed to select genes for pathway analysis: (1) genes with germline mutations in ≥ 2 patients were selected, except that all genes in the luminal B/Her2- subtype were included; (2) genes with somatic mutations in at least 2 patients or in ≥ 10% of the patients (whichever is higher), except that all genes in the luminal B/Her2- subtype were included; and (3) for the whole cohort pathway analysis, the list of genes with germline mutations in ≥ 2 patients and the list of genes with somatic mutations in at least 5% of the total patients were combined to be used as the input. The DAVID 6.8 webserver was used for pathway analysis and *Homo sapiens* was selected as the background organism. KEGG pathways with *p*-values of less than 0.05 were selected as enriched pathways in EOBC patients.

### 4.11. Ethics Approval and Consent to Participate

All human materials used in the study for either WGS or WES analysis were reviewed and approved by the Human Subject Research Ethics Committee/ Institution Review Board (IRB) of the Academia Sinica (Approval number: AS-IRB02-99065), NTUH and the ISCTCR, Chang Gung Memorial Hospital. All participating patients provided their clinical information and informed consent for the research. All regulatory guidelines were strictly complied with throughout the study.

### 4.12. Availability of Data and Material

Key genomic aberrations and clinical data are provided in supplementary files. The BAM files generated and/or analyzed during current study are available in NCBI with BioProject ID: PRJNA574486. Somatic mutation and germline mutation data are available in our website at https://topsciencebiotech.com/main/eobc/.

## 5. Conclusions

We performed comprehensive analysis of the EOBC mutation spectrum in Taiwanese women. Our study highlighted distinguishable molecular features of EOBC and non-EOBC patients by incorporating other breast cancer datasets. Further, we identified diverse mutation profiles among molecular subtypes. Our results indicated that mutations in the genes of ABC transporters and ECM–receptor interaction, focal adhesion and PI3K–Akt-signaling pathways could be potential therapeutic targets. Additionally, somatic mutations alone may not be sufficient to characterize EOBC disease, while germline mutations in structural proteins may also act as a causal factor to initiate EOBC tumorigenesis. Functional characterization of key mutations may provide further insights into the mechanism of cancer development to improve clinical outcomes.

## Figures and Tables

**Table 1:** Taiwanese early onset breast cancer (EOBC) cohort structure and associated sequencing methods.

Category	Description	Number of Patients	WES	WGS
Subtype	Her2+	9	8	1
	Luminal A	34	34	0
	Luminal B/Her2+	20	18	2
	Luminal B/Her2-	14	13	1
	Triple negative	13	13	0
Age group	< 37 (median)	39	37	2
	≥ 37	51	49	2
Stage group	Ia, Ib	15	13	2
	IIa, IIb	42	41	1
	IIIa, IIIb, IIIc	24	23	1
	IVa, IVb	5	5	0
	Unknown	4	4	0
Family history	No family history	79	75	4
	With family history	11	11	0

WES—whole-exome sequencing; WGS—whole-genome sequencing.

**Table 2:** Subtype-based pathway analysis.

Pathway ID	Pathway	Her2+	Luminal_A	Luminal B Her2+	Luminal B Her 2-	Triple Negative	Whole Cohort
* hsa05222	Small cell lung cancer	0.001	0.009	0.032	0.006	0.020	0.014
hsa04380	Osteoclast differentiation	0.006	–	–	–	–	–
* hsa05146	Amoebiasis	0.021	0.018	–	–	–	–
* hsa05200	Pathways in cancer	0.022	–	–	0.012	–	–
hsa04919	Thyroid hormone-signaling pathway	0.026	–	–	–	–	–
hsa04510	Focal adhesion	0.028	0.013	0.009	0.0001	0.051	0.004
hsa04071	Sphingolipid-signaling pathway	0.030	–	–	–	–	–
hsa04512	ECM–receptor interaction	–	0.001	0.008	0.007	–	0.001
* hsa05016	Huntington’s disease	–	0.010	–	–	–	0.031
hsa04151	PI3K–Akt-signaling pathway	–	0.016	0.009	0.001	–	0.032
* hsa05213	Endometrial cancer	–	–	0.006	0.021	0.048	–
hsa02010	ABC transporters	–	–	0.024	0.030	–	0.00004
hsa03460	Fanconi anemia pathway	–	–	0.039	–	–	–
hsa04015	Rap1-signaling pathway	–	–	–	0.0004	–	–
hsa05230	Central carbon metabolism in cancer	–	–	–	0.002	–	–
* hsa05218	Melanoma	–	–	–	0.014	–	–
* hsa05215	Prostate cancer	–	–	–	0.020	–	–
hsa04060	Cytokine–cytokine receptor interaction	–	–	–	0.024	–	–
hsa04923	Regulation of lipolysis in adipocytes	–	–	–	0.030	–	–
* hsa05412	Arrhythmogenic right ventricular cardiomyopathy (ARVC)	–	–	–	0.037	–	–
hsa04520	Adherens junction	–	–	–	0.037	–	–
hsa05205	Proteoglycans in cancer	–	–	–	0.043	–	–
* hsa04930	Type II diabetes mellitus	–	–	–	0.043	–	–
hsa04611	Platelet activation	–	–	–	0.048	–	–
* hsa05210	Colorectal cancer	–	–	–	0.049	–	–
hsa04630	Jak–STAT-signaling pathway	–	–	–	0.050	–	–
hsa04530	Tight junction	–	–	–	–	–	0.008
hsa04974	Protein digestion and absorption	–	–	–	–	–	0.017

* indicates pathway related to diseases that may not be breast cancer.

## Supplementary Materials

The following are available online at https://www.mdpi.com/2072-6694/12/8/2089/s1, Supplementary Figure S1: Validation of somatic mutations by Sanger sequencing, Supplementary Figure S2: Number of genes affected by somatic mutations among patients in Taiwanese EOBC cohort, Supplementary Figure S3: Comparison of top 10 genes with somatic mutations in Taiwanese EOBC cohort to the individual EOBC (A) and individual non-EOBC (B) groups in each external cohort, Supplementary Figure S4: Kaplan Meier disease/progression-free survival plot for pooled EOBC and pooled non-EOBC patients from external cohorts, Supplementary Figure S5: Common and specific germline mutations in sisters with a family history of breast cancer, Supplementary Table S1: Library statistics, Supplementary Table S2: Characterization of somatic mutations, Supplementary Table S3: Clinical information and somatic mutations of all Taiwanese EOBC patients (As of May, 2017), Supplementary Table S4: Subtype-based top genes with somatic mutations, Supplementary Table S5: Description of non-Taiwanese breast cancer cohorts used for comparison, Supplementary Table S6: Differentially mutated genes in pooled EOBC and pooled non-EOBC of external cohorts, Supplementary Table S7: Copy number variations in different cytobands by subtype, Supplementary Table S8: Overlap in CNV regions between sisters 7768 and 7942.
