# Supplementary material for: Comprehensive Cohort Analysis of Mutational Spectrum in Early Onset Breast Cancer Patients
Source: Cancers (Basel). 2020 Jul 28;12(8):2089. doi: 10.3390/cancers12082089 (PMC7464007; doi:10.3390/cancers12082089)
Supplement: Supplementary file 1 [file cancers-12-02089-s001.zip › Supplementary figures.docx]

**Comprehensive cohort analysis of mutational spectrum in early-onset breast cancer patients**

Mohit K. Midha^1, 2^, Yu-Feng Huang^1^, Hsiao-Hsiang Yang^1^, Tan-Chi Fan^3^, Nai-Chuan Chang^3^, Tzu-Han Chen^1^, Yu-Tai Wang^4^, Wen-Hung Kuo^5^, King-Jen Chang^5^, Chen-Yang Shen^6^, Alice L. Yu^3, 7^, Kuo-Ping Chiu^1, 2, 8^* and Chien-Jen Chen^1^

^1^Genomics Research Center, Academia Sinica, Taipei, Taiwan

^2^Institute of Biochemistry and Molecular Biology, National Yang-Ming University, Taipei, Taiwan

^3^Institute of Stem Cell and Translational Cancer Research, Chang Gung Memorial Hospital at Linkou and Chang Gung University, No. 5, Fu-Shin St., Kuei Shang, Taoyuan 333, Taiwan

^4^National Center for High-Performance Computing, Hsinchu Science Park, Taiwan

^5^Department of Surgery, National Taiwan University Hospital, Taipei, Taiwan

^6^Institute of Biomedical Sciences, Academia Sinica, Taipei, Taiwan

^7^Department of Pediatrics, University of California in San Diego, CA, USA

^8^Department of Life Sciences, College of Life Sciences, National Taiwan University, Taipei, Taiwan

*Corresponding Author:

Kuo-Ping Chiu, Ph.D.

Genomics Research Center, Academia Sinica

128 Academia Road, Sec. 2, Nankang District, Taipei 115, Taiwan

Tel: (886) 2-2787-1257 | Fax: (886) 2-2789-9924

Email: [chiukp@gate.sinica.edu.tw](mailto:chiukp@gate.sinica.edu.tw)


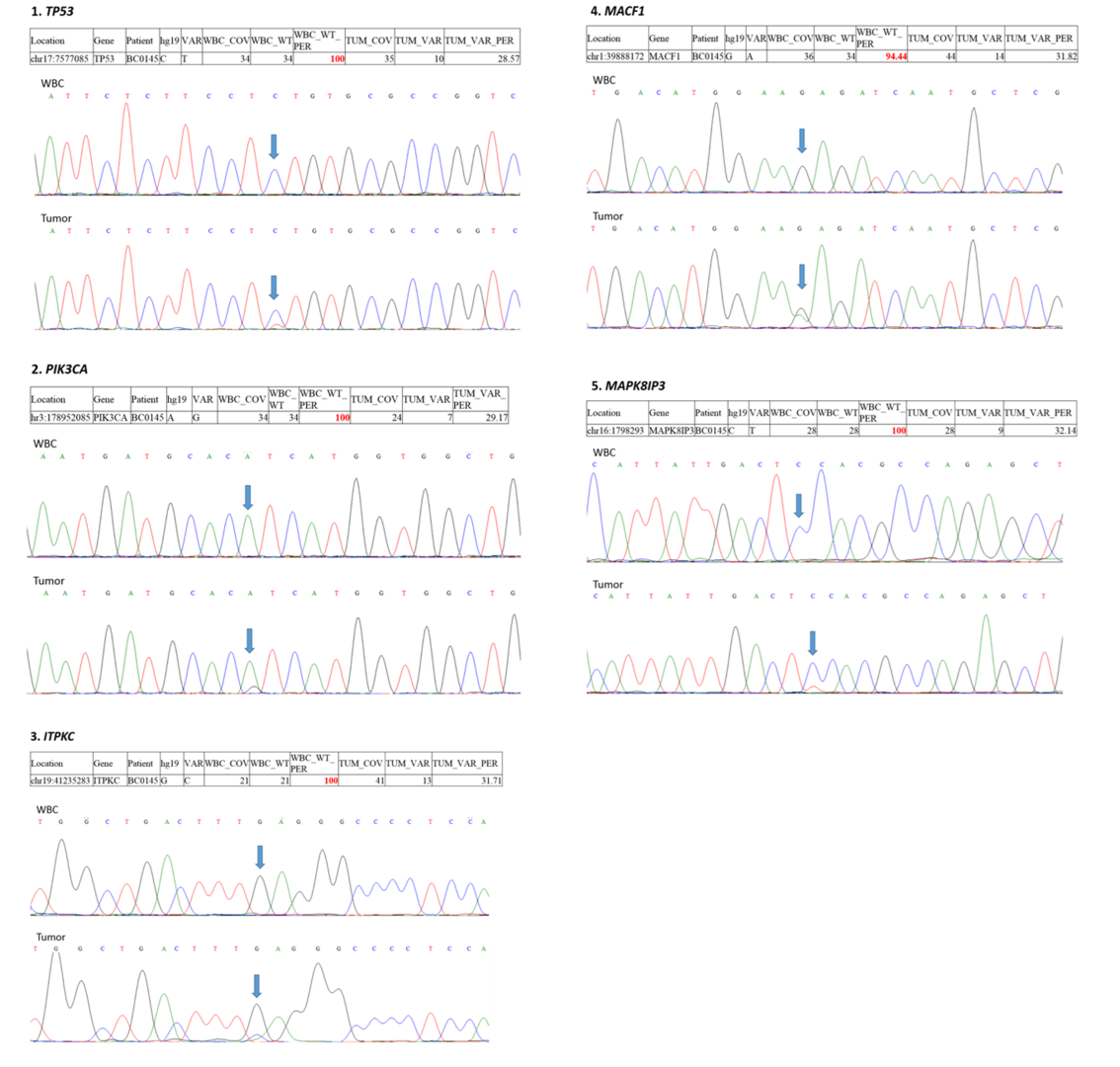


**Supplementary Figure 1. Validation of somatic mutations by Sanger sequencing.** Somatic missense mutations in *TP53*, *PIK3CA* and three other genes randomly selected from the WBC and tumor tissue samples of patient BC0145 were validated by Sanger sequencing. Empirically, PCR fragments generated with specific PCR primer pairs flanking the mutation sites were cloned into the pZBack vector, which was used to transform *E. coli* DH5-Alpha competent cells which were then cultured in agar plates. Colonies were picked from the plates and cultured in aqueous media. Plasmids were isolated in mini-preps and sequenced by Sanger sequencing. Ten bases on each side of the mutation in the chromatogram are shown together with the mutated sites.


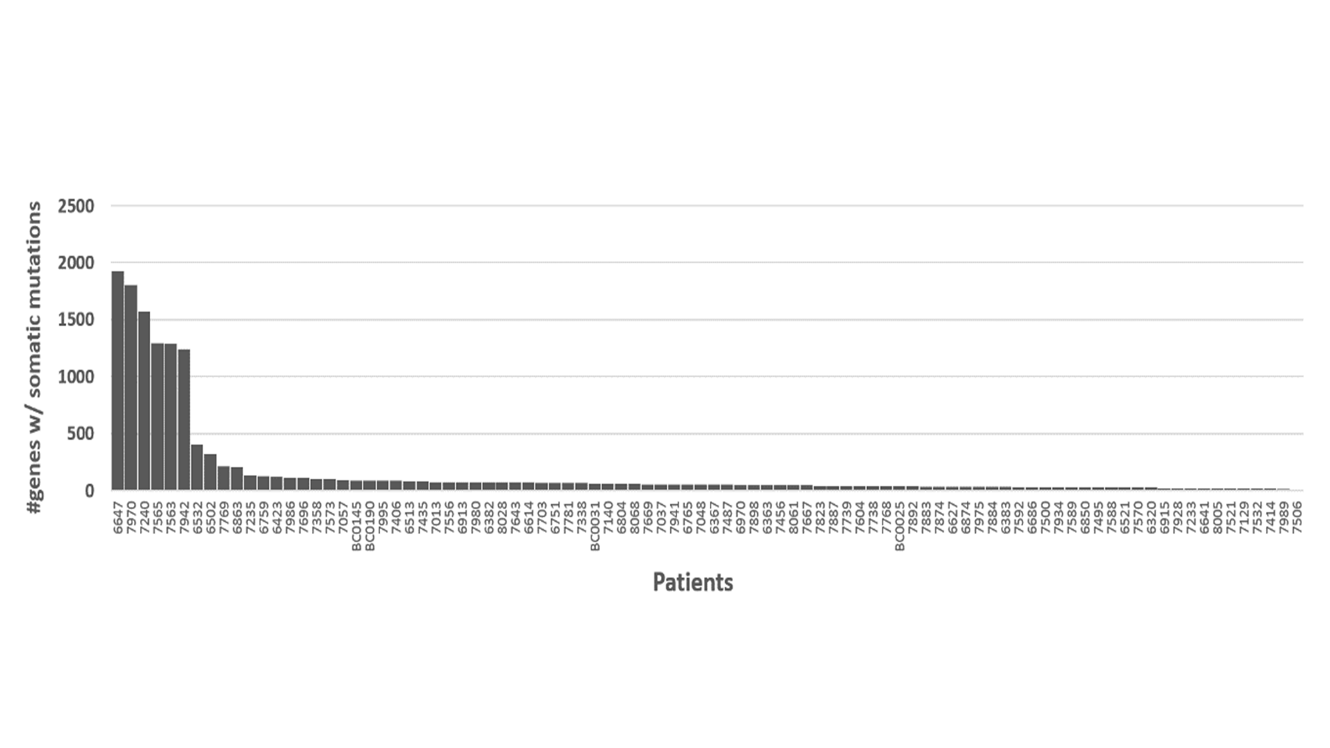


**Supplementary Figure 2. Number of genes affected by somatic mutations among patients in Taiwanese EOBC cohort.** Patients are sorted on the number of mutated genes.


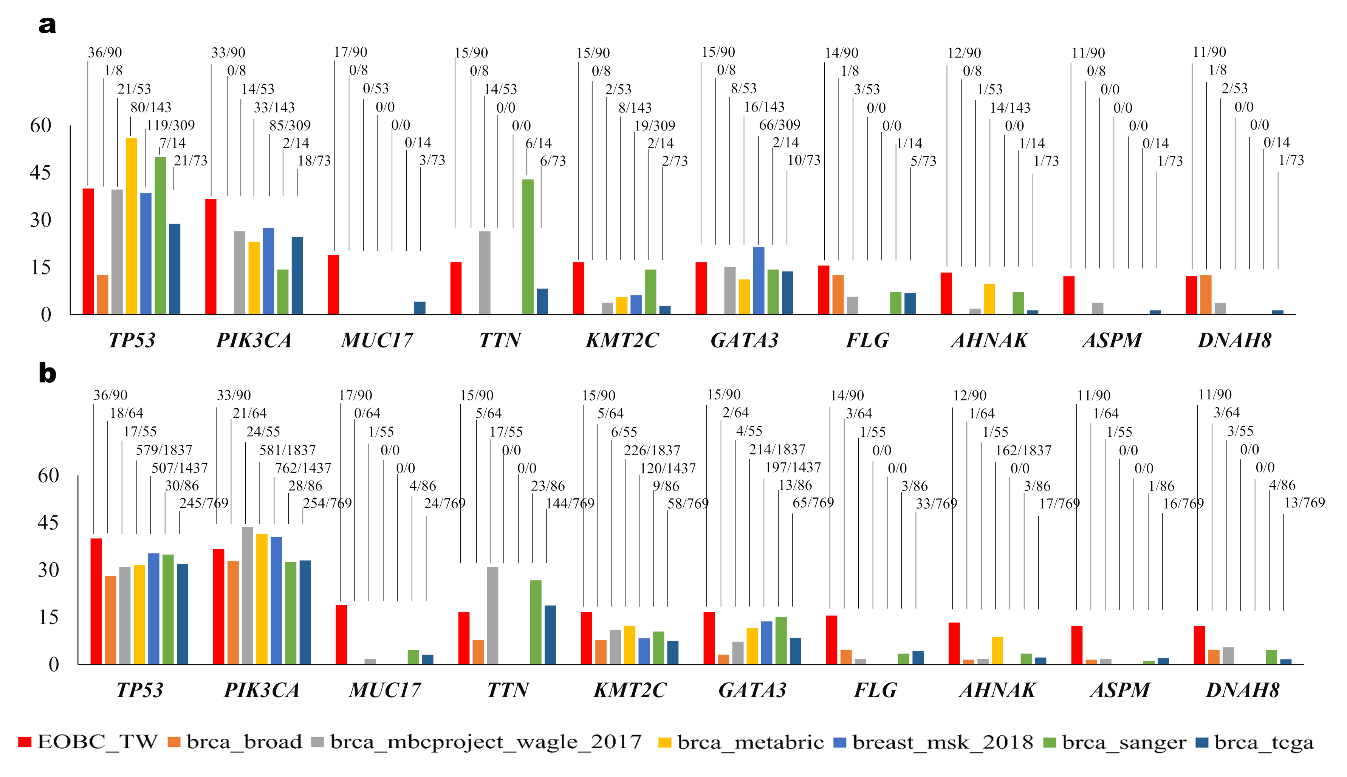


**Supplementary Figure 3. Comparison of top 10 genes with somatic mutations in Taiwanese EOBC cohort to the individual EOBC (A) and individual non-EOBC (B) groups in each external cohort.** Notice that Taiwanese cohort has only EOBC data and, thus, the heights of red bars in two panels for each gene retain the same. Numbers corresponding to each vertical bar for each gene show number of patients with mutation in that particular gene over total number of patients sequenced for that gene.


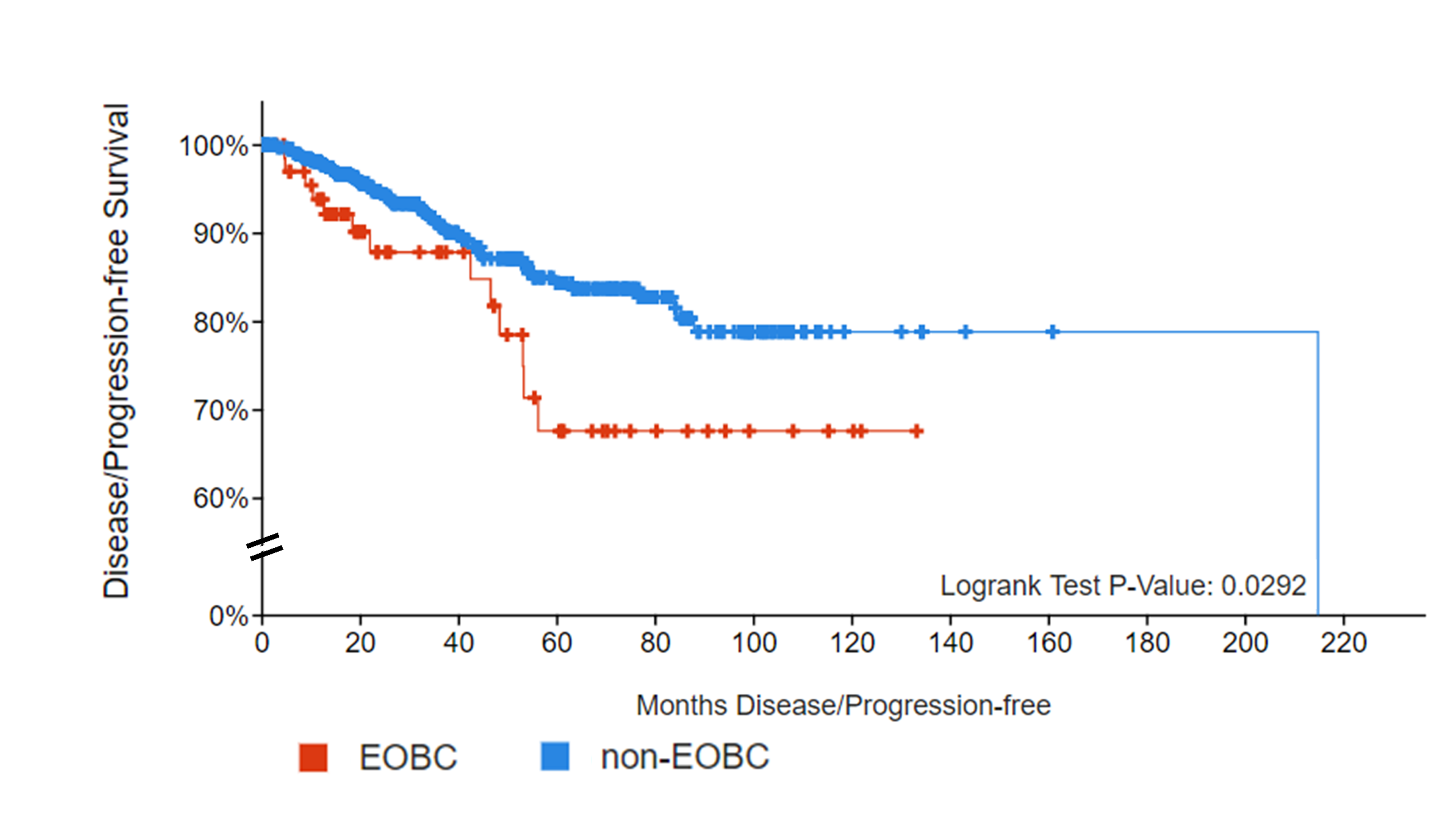


**Supplementary Figure 4. Kaplan Meier disease/progression-free survival plot for pooled EOBC and pooled non-EOBC patients from external cohorts.** Disease/progression-free survival analysis was performed for pooled EOBC (70 patients) and pooled non-EOBC (714 patients) over a period of 220 months.


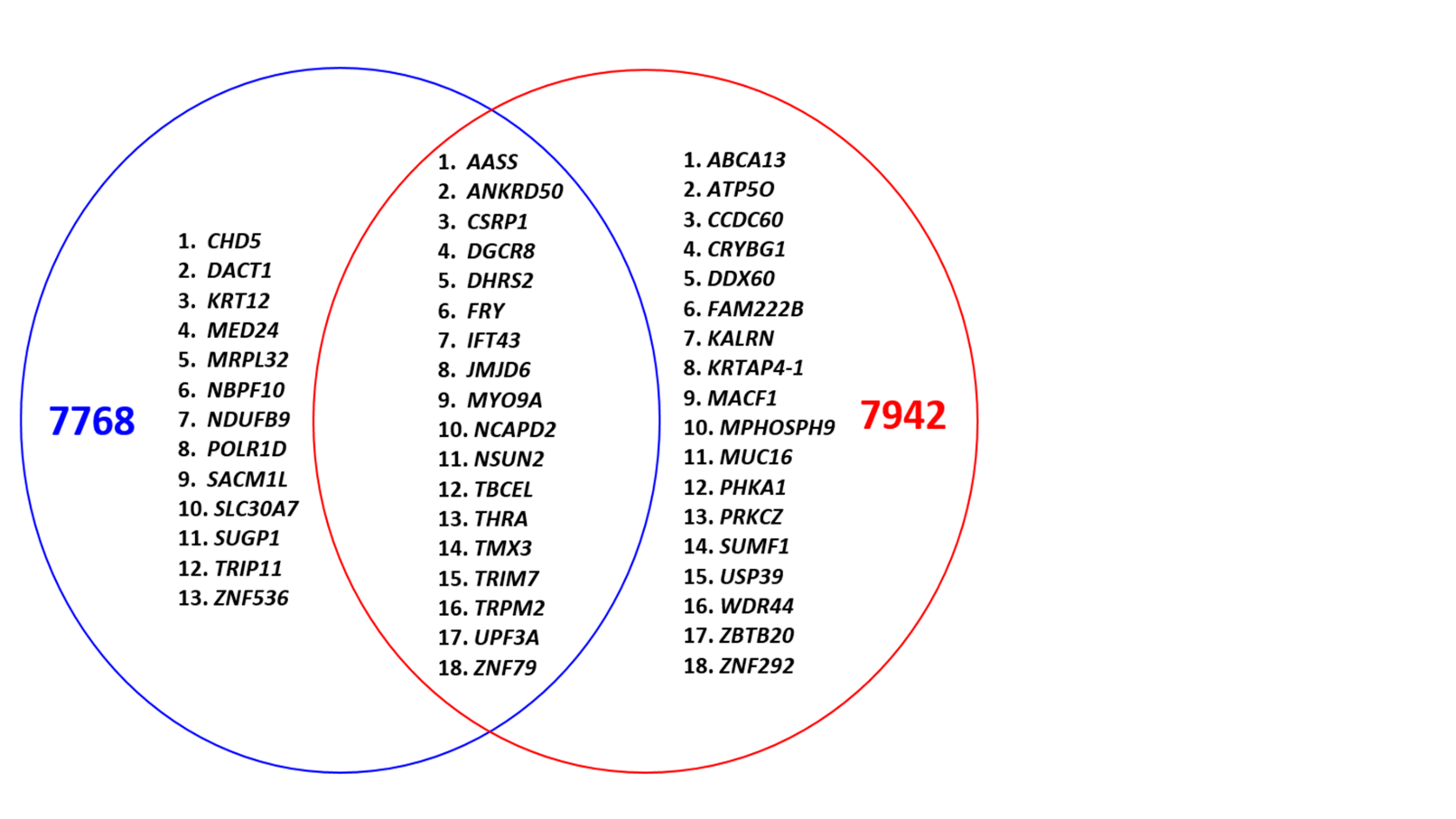


**Supplementary Figure 5. Common and specific germline mutations in sisters with a family history of breast cancer**. Germline mutations in sisters (7768 and 7942) were identified and split into two categories: common and individual-specific.

**Supplementary Table captions**

**Supplementary Table 1.** Library statistics.

**Supplementary Table 2.** Characterization of somatic mutations.

**Supplementary Table 3.** Clinical information and somatic mutations of all Taiwanese EOBC patients (As of May, 2017).

**Supplementary Table 4.** Subtype-based top genes with somatic mutations.

**Supplementary Table 5.** Description of non-Taiwanese breast cancer cohorts used for comparison.

**Supplementary Table 6.** Differentially mutated genes in pooled EOBC and pooled non-EOBC of external cohorts.

**Supplementary Table 7.** Copy number variations in different cytobands by subtype.

**Supplementary Table 8.** Overlap in CNV regions between sisters 7768 and 7942.
